# Controlling Infectious Risk in Transfusion: Assessing the Effectiveness of Skin Disinfection in Blood Donors

**DOI:** 10.3390/healthcare10050845

**Published:** 2022-05-05

**Authors:** Antonella Arghittu, Marco Dettori, Grazia Maria Deriu, Serena Soddu, Pietro Carmelo Manca, Anna Angela Carboni, Irene Collu, Alessandra Palmieri, Giovanna Deiana, Antonio Azara, Paolo Castiglia, Maria Dolores Masia

**Affiliations:** 1 Department of Biomedical Sciences, University of Sassari, 07100 Sassari, Italy; arghittu.antonella@gmail.com (A.A.); ireneulloc@hotmail.it (I.C.); giovanna.deiana90@gmail.com (G.D.); 2 University Hospital of Sassari, 07100 Sassari, Italy; mariagrazia.deriu@aousassari.it (G.M.D.); serena.soddu@aousassari.it (S.S.); pietro.manca@aousassari.it (P.C.M.); annaangela.carboni@aousassari.it (A.A.C.); luca@uniss.it (A.P.); azara@uniss.it (A.A.); castigli@uniss.it (P.C.); 3 Department of Medical, Surgical and Experimental Sciences, University of Sassari, 07100 Sassari, Italy; mdmasia@uniss.it

**Keywords:** skin disinfection, blood donors, bacterial contamination, transfusion risk

## Abstract

Bacterial infectious risk is a major problem in transfusion medicine. The type of micro-organisms isolated during bacterial contamination of blood products indicates that the donor’s skin is its main source. In this context, the primary measures to reduce this risk are: (a) optimal disinfection of the donor’s arm and (b) satellite bag diversion of the initial volume of blood collected. This work aimed to verify the effectiveness of skin disinfection of the blood donor’s venipuncture site. Two methodological approaches were used: (a) qualitative and quantitative microbiological testing of the skin at the collection site, before and post-disinfection; (b) qualitative microbiological testing of the first deviated blood. Pre-disinfection testing showed skin microbial load values between 3 and >200 CFU/plate. More than two-thirds of the isolates were Gram-positive bacteria (77.8%) of which 57.7% were *staphylococci*. Among Gram-negative bacteria, *Pseudomonadaceae*, *Enterobacteriaceae,* and *Acinetobacter* spp. were isolated from the blood donors (BDs). Post-disinfection, a 100% reduction in microbial load was observed in 84.4% of BDs. Microbiological testing of the first blood diverted sample revealed the presence of microbial flora in 1.9% samples; of the isolates, 83.3% were non-aureus *staphylococci*. This study highlights the importance of the correct application of skin disinfection procedures in order to ensure blood safety.

## 1. Introduction

The transfusion of blood and blood components is an essential and irreplaceable therapeutic procedure in the treatment of many pathological conditions. However, like other therapies, it is not without risks of various types. Such risks relate to (i) genetic difference of the transfused blood; (ii) the possibility of adverse events deriving from an interaction between the medical intervention and the individual’s organic equilibrium; (iii) medical error; (iv) the technical and/or administrative errors due to the mistaken application of procedural rules; (v) the possibility of transmission of both infectious and non-infectious diseases [1,2,3,4].

With regard to the risk of transmitting infectious diseases, several strategies, including donor screening and deferral, blood testing, and pathogen inactivation, have resulted in all-time low levels, particularly in developed countries. Nevertheless, while lower than in the past, the bacterial infection risk is still a major problem in transfusion medicine [5,6,7,8,9,10].

Bacterial contamination of blood products can be endogenous in origin, mainly transient donor bacteraemia due to minor chronic infections, asymptomatic infections, and dental procedures, or exogenous due to inadequate disinfection of the skin at the venipuncture site, improper venipuncture technique, presence of a skin tissue plug due to needle insertion, plastic defects in the collection bag, bacterial contamination of anticoagulant and disinfectant, and contamination of the venipuncture needle by the external environment [1,5].

The type of micro-organisms isolated during bacterial contamination indicates that the main source is the donor’s skin [1,5,10,11,12,13,14]. Although under physiological conditions, the skin constitutes a valid barrier to the penetration of micro-organisms into the underlying tissues, any injury that compromises its structural integrity (wounds, needles, catheters, etc.) may constitute a potential entry point for micro-organisms. In this sense, blood sampling is a critical moment during which bacteria present on the skin can pass into the collection system through the phlebotomy needle, through a skin plug punched out by the needle, or through the small skin flaps produced by the needle itself during phlebotomy [15,16,17,18,19,20,21].

In this context, the primary measures to reduce the risk of bacterial contamination of blood products are: (a) optimal disinfection of the donor’s arm to reduce skin microbial load and (b) satellite bag diversion of the initial volume of blood collected in order to remove the first aliquot of donor blood, which may have higher concentrations of skin flora [19,20,22,23].

As several variables can affect the risk of bacterial contamination of the donation product (e.g., type, concentration, and application method of the disinfectant used; contact time of the disinfectant with the skin), the aim of this work was to verify the effectiveness of skin disinfection of the blood donor’s venipuncture site. This study was included in a wide-ranging program of clinical risk surveillance and quality control of care that the Hygiene and Hospital Infection Control Unit of the University Hospital of Sassari (AOU-SS) carries out at the same University Hospital [24,25,26,27,28,29,30].

## 2. Materials and Methods

### 2.1. Study Setting

The study was carried out by the operators from the Hospital Hygiene and Infection Control Unit of the AOU-SS at the immunohaematology and transfusion medicine service of the same hospital. This service has an annual average of approximately 8100 donors, 10,380 donations, and 21,850 blood components produced. In accordance with the regulations in force, the age of donors is between 18 and 65, and the act of donating is voluntary and unpaid.

### 2.2. Assessing Skin Disinfection

In Italy, the National Blood Centre, given Legislative Decree no. 208 of 9 November 2007, issued its ‘Guidelines for the prevention of bacterial contamination of whole blood and blood components’. In addition to reiterating the procedures for skin disinfection, these guidelines provide for the sole use of collection systems that enable the deviation of the first volume of blood from the donation to be used for the performance of the diagnostic tests laid out in the ministerial provisions [31,32]. Because of this, the effectiveness of skin disinfection was assessed in this study during a 5 year period on at least 1% of the donations, using two different approaches: (a) directly, by qualitative and quantitative microbiological testing of the skin at the collection site, before and after disinfection (donor arm testing); (b) indirectly, by qualitative microbiological testing of the portion of blood destined for the satellite bag (first diverted blood); this blood was taken from donors different from group a. In both groups, the donors or the donation product (first diverted blood) were consecutively selected; first the donation products, and subsequently, the donors from those present at the time of testing.

### 2.3. Qualitative and Quantitative Microbiological Testing of Skin

BDs were recruited after medical screening and a brief explanation of the purpose of the study. Microbiological testing of the donor skin was performed immediately before and after disinfection of the skin in the venipuncture site. Disinfection was carried out according to protocol using chlorhexidine 2%. For this purpose, 55 mm diameter RODAC (replicate organism detection and counting) contact plates containing plate count agar (PCA) medium with a neutralising agent (Tween 80) to inhibit the residual activity of the disinfectant were placed on the blood sampling area, exerting constant pressure for 40 s. The plates were then incubated in an incubator at 36 ± 1 °C for 48 h. After this time, the microbial load was determined, the micro-organisms isolated were identified, and for *staphylococci* only, the sensitivity to methicillin was tested. With regard to microbial load determination, when the number of colonies grown was such that they almost completely or completely covered the surface of the culture medium, the result of the count was expressed as >200 CFU/plate [33].

The efficacy of skin disinfection was assessed both quantitatively and qualitatively.

With regard to the quantitative evaluation, the efficacy of skin disinfection was calculated as the percentage of CFU reduction in post-disinfection vs. CFU detected in pre-disinfection. In particular, the reduction was calculated as 1 − (X_CFU_ post/X_CFU_ pre) ∗ 100, where X_CFU_ post is the number of CFU observed post-disinfection, and X_CFU_ pre is the number of CFU observed before the skin disinfection.

As regards the qualitative evaluation, the proportion of non-effectiveness (i.e., positivity with at least 1 CFU/plate post-disinfection) was determined for each quartile of subjects observed sorted by pre-disinfection skin microbial load.

As the qualitative and quantitative characterisation of the microbial flora present on the skin in the venipuncture site prior to disinfection is instrumental in identifying individual and environmental factors that may influence the varying degree of skin contamination, and possibly, the efficacy of disinfection itself, the results on the microbial flora present pre-disinfection on donors’ skin were processed according to the variables sex, age, and season.

### 2.4. Qualitative Microbiological Testing of the First Portion of Diverted Blood Intended for Satellite Bag

Testing of the first diverted blood was performed using the BACTEC 9240 automated blood culture system (Becton Dickinson Company, Franklin Lakes, NJ, USA; http://legacy.bd.com/ds/technicalCenter/clsi/clsi-9000bc2.pdf, accessed on 22 April 2022). Under aseptic conditions, two aliquots of blood destined for the satellite bag (approximately 10 mL each) were inoculated into the anaerobic culture bottle (the first) and the aerobic culture bottle (the second). The inoculated culture bottles were placed in the BACTEC 9240 system for incubation at 35 ± 1.5 °C with continuous agitation. This system tracks the amount and rate of CO_2_ production, indicative of microbial growth. Culture bottles that produced a positive result were subcultured onto blood agar plates to isolate microbial colonies for identification and methicillin susceptibility testing (*staphylococci* only). Culture bottles in which no growth was detected were removed from the automated system after 7 days, according to the manufacturer’s instructions.

### 2.5. Statistical Analysis

Data were entered on Excel (Microsoft Office, Microsoft Corporation, Redmond, WA, USA) and analysed using the STATA software 16.1 (StatCorp., Austin, TX, USA). Quantitative variables were expressed as the mean ± standard deviation and range or as median and interquartile range (IQR), categorical variables as proportions.

The difference among groups as regards the microbial load was tested using the rank-based nonparametric Kruskal-Wallis H test (KW). The relationship between variables was calculated using the kernel nonparametric regression model. The independent variables (sex and season) were categorised in ascending order based on the average bacterial load values recorded.

Data were sorted according to pre-disinfection skin microbial load and divided into quartiles. The first quartile (i.e., 25th percentile) and the third quartile (i.e., 75th percentile) were calculated and are indicated in tables as Q1 and Q3, respectively. The qualitative evaluation of the efficacy of disinfection in relation to skin microbial load was performed using trend analysis for proportions.

Prevalence and 95% confidence intervals were reported as percentages. Differences among proportions were tested with the z-test. A two-tailed *p*-value of less than 0.05 was considered statistically significant.

## 3. Results

The adequacy and effectiveness of the disinfection procedure was assessed (a) directly, by microbiological testing of the skin in the blood collection area of 330 donors immediately before and after disinfection, and (b) indirectly, by testing the first blood diverted in 267 satellite bags.

### 3.1. Microbiological Testing of Skin before and after Antisepsis

A total of 330 donors aged 18–65 years were recruited (mean age 45.3 ± 11.4; median age 46; IQR 17), of whom one-third (110/330; 33.3%) were aged 46–55 years. The prevailing sex was male (229/330; 69.4%). Of the tests, 26.4% (87/330) were performed in winter (January–March). The distribution of BDs by age group, sex, and season is shown in Table 1.

Pre-disinfection testing showed skin microbial load values between 3 and >200 CFU/plate (median 47; IQR 60), the latter being observed in 15.8% of donors (52/330). The distribution of microbial load values showed no significant difference between age groups (KW: chi-squared 171 = 4.148; *p* = 0.386) but the highest median values were in the 26–35 age group (median 57; IQR 56.25) and the lowest in the 36–45 age group (median 41; 170 IQR 56) (Table 2). In relation to the seasons, the difference in the microbial load detected was not statistically significant (KW: chi-squared = 2.798; *p* = 0.4239) (Table 2).

When analysing the microbial load values both by age group and season, the highest median values were observed in spring in the age group 26–35 and in winter in the age group 46–55 (Table 2).

The microbial load values in male donors ranged from 3 CFU/plate to >200 CFU/plate (median value 46 CFU/plate; IQR 60), without statistically significant differences between age groups (KW: chi-squared = 2.698; *p* = 0.609) (Table 3). In female donors, the same values ranged from 4 CFU/plate to >200 CFU/plate (median value 47 CFU/plate; IQR 68), without statistically significant differences among age groups (KW: chi-squared = 2.747; *p* = 0.601) (Table 3). The median values of microbial load between sexes, 46 and 47 CFU/plate for male and female donors, respectively, did not differ statistically significantly (KW: chi-squared = 0.058; *p* = 0.81).

Overall, the distribution by sex, age, and season showed the highest median values in male donors in spring in the 26–35 age group and in female donors in spring in the 18–25 age group and in winter in the 56–65 age group, where in one female donor each season, a microbial load >200 CFU/plate was recorded.

The results of the nonparametric kernel regression analysis to assess the association between the bacterial load found and the dependent variables (sex, age, and season) are shown in Table 4.

As Table 4 shows, the bacterial load increased with age and female sex, with differences between seasons, although not statistically significant.

**Figure 1:**
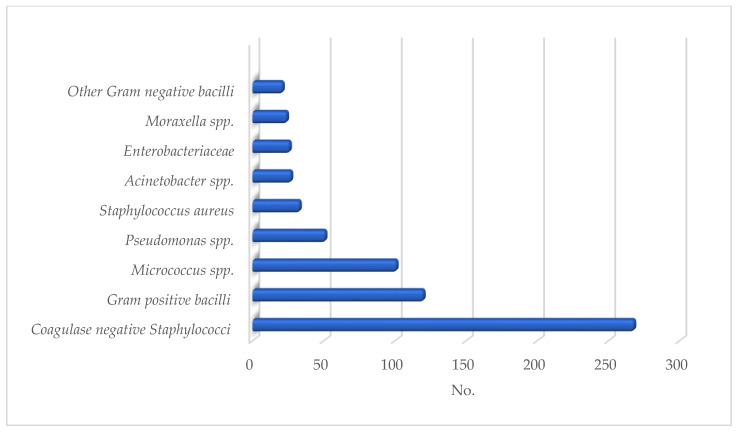
Qualitative aspects of pre-disinfection skin microbial contamination.

Qualitatively, more than two-thirds of the isolates (522/671) were Gram-positive bacteria (77.8%; 95% CI: 77.5–81.0), of which 57.7% (301/522; 95% CI: 53.5–61.9) were *staphylococci*. Additionally, 11% (33/301; 95% CI: 7.5–14.5) of the *staphylococci* were *Staphylococcus aureus*, of which 9.1% (3/33; 95% CI: 7.1–18. 9) were methicillin-resistant (methicillin-resistant *Staphylococcus aureus*, MRSA); methicillin resistance was also detected in 3.4% (9/268; 95% CI: 1.2–5.6) of the non-*aureus* (coagulase-negative) *staphylococci*. Among Gram-negatives, the most frequently isolated micro-organisms were *Pseudomonadaceae* (51/149; 34.2%; 95% CI: 43.0–59.0) (Figure 1).

Efficacy was assessed both as a percentage of load reduction pre- and post-disinfection and in relation to initial load values.

As regards the percentage of reduction, tests carried out after disinfection showed a 100% reduction in microbial load in 280 out of 330 donors (84.8%; 95% CI: 80.9–88.7); the remaining 50 donors (15.2%; 95% CI: 11.3–19.1) had microbial load values between 1 and 28 CFU/plate, with a percentage reduction in microbial load ranging from 71.9% to 99.5% (mean percentage reduction 93.7% (95% CI: 86.9–100.0)).

The efficacy of skin disinfection was evaluated in relation to the pre-disinfection microbial load (Table 5).

As the table shows, the proportion of non-effectiveness increased significantly in accordance with the microbial load found pre-disinfection.

Among the micro-organisms isolated, 91.5% (54/59; 95% CI: 84.4–98.6) were Gram-positive, predominantly *staphylococci* (35/59; 59.3% of the total; 95% CI: 46.8–71.8), all coagulase-negative, of which 1 (2.9%) was methicillin-resistant. Among the 5 Gram-negative isolates, there was an *Enterobacteriaceaea* (*Proteus* spp.).

### 3.2. Microbiological Testing of First Diverted Blood

Testing was performed on 267 donations. Microbial flora was detected in five samples (1.9%; 95% CI: 0.3–3.5); 83.3% of the isolates were non-aureus *staphylococci*, all of which were methicillin-sensitive.

## 4. Discussion

Microbiological testing of the skin prior to disinfection revealed a bacterial colonisation of the skin that varied both quantitatively, with microbial load values between 3 and >200 CFU/plate, and to a lesser extent, qualitatively, with different bacterial species isolated. In addition to the more common skin flora, *Staphylococcus aureus*, *Enterobacteriaceae*, and *Pseudomonadaceae* were isolated. These micro-organisms, especially *Enterobacteriaceae* and *Staphylococcus aureus*, are considered to be among the most undesirable contaminants of blood products on the basis of clinical observations and their virulence characteristics [34,35,36]. In addition, some methicillin-resistant strains of *staphylococci* have been reported, confirming their presence in hospital and community settings [37,38,39].

The analysis of skin microbial load values by sex, age, and season of donation, independently for each variable, indicated that female donors, the 26–35 age group, and the autumn and winter seasons were the most critical factors.

Conversely, other authors have observed that intrinsic factors, such as sex and age, and extrinsic factors, such as environmental characteristics (e.g., climate), can influence the quality and quantity of the skin microbial flora, so that the highest values can likely be expected in men (physiological sex differences affecting skin properties include hormone production, sweating rate, sebum production, surface pH, skin thickness, etc.), summer (higher exposure and skin moisture), and older age groups, as it is known that surface flora increases with age [12,15,18,40,41]. Our findings did not confirm these figures. However, the differences we observed were not significant.

The disinfection resulted in a reduction in the baseline microbial load ranging from 71.9% to 100%, the latter observed in approximately 85% of donors. Even if the probability of non-effectiveness was significantly associated with the extent of baseline contamination but not with the type of antiseptic used (always chlorhexidine 2%), the variability observed may have been influenced by human factors, i.e., the operator performing antisepsis. An optimal disinfection also depends on the way in which the disinfectant is applied. The positive result of the microbiological tests on the first blood diverted confirmed the importance of this procedure, since even after disinfection was performed, the possibility of contamination of the blood remained (we found it in 1.9% of the cases). Although careful antisepsis can significantly reduce the amount of micro-organisms present on the superficial layers of the skin, it cannot act as effectively on the deeper layers [32,42]. Therefore, the collection bag can be contaminated when the needle is inserted [17]. However, the absence of contamination in more than 98% of the tests confirmed the indispensability of an effective and properly performed skin disinfection.

## 5. Conclusions

The donor plays a major role in transfusion. If on the one hand, they provide the product to be transfused, on the other hand, they are required to ensure that this product is as safe as possible. In this context, the adoption of correct lifestyles and the willingness to exclude oneself in the event of risky conditions and/or behaviour, alongside attention to one’s personal hygiene, are of paramount importance. Careful cleaning of the donor (especially the arm) and clothing can lead to a reduction in skin contamination, thereby also favouring the effectiveness of the disinfection procedure. Our results highlighted that a possible failure of disinfection depends on the initial load.

The disinfection procedure plays a crucial role in preventing the risk of bacterial contamination of blood donations and must be carried out with the utmost care on each donor, even the last of a successful day’s donations. The choice of disinfectant and the duration of its application are essential factors that can reduce the risk of bacterial contamination from the skin flora, and as such, the healthcare worker who takes the blood sample must also adopt suitable behaviours that favours the effectiveness of disinfection, which include carefully washing hands before the procedure and respecting the timing and method of application of the disinfectant.

It is with this in mind that awareness-raising and training activities should be directed towards healthcare workers—useful in all clinical risk training contexts—and towards those citizens who, through a voluntary and unpaid act, ensure the availability of that most precious commodity, blood.

## Figures and Tables

**Table 1:** Distribution of BDs by age group, sex, and season.

Age Groups	Winter			Spring			Summer			Autumn			Total for Age Groups
	BDs	M	F	BDs	M	F	BDs	M	F	BDs	M	F	BDs
**18–25**	4	4	0	5	4	1	6	5	1	6	2	4	21
**26–35**	15	9	6	11	8	3	13	8	5	11	5	6	50
**36–45**	23	15	8	18	14	4	16	11	5	26	18	8	83
**46–55**	28	20	8	28	21	7	29	21	8	25	16	9	110
**56–65**	17	16	1	16	10	6	17	13	4	16	9	7	66
**Total for season**	87	64	23	78	57	21	81	58	23	84	50	34	330

BDs = Blood Donors; M = Male; F = Female.

**Table 2:** The distribution of microbial load before skin disinfection in different age groups and seasons.

Age Groups	Winter	Spring	Summer	Autumn	Total for Age Groups
	CFU Median (Q1–Q3)	CFU Median (Q1–Q3)	CFU Median (Q1–Q3)	CFU Median (Q1–Q3)	CFU Median (Q1–Q3)
**18–25**	43 (31.25–52.75)	78 (42–>200)	42.5 (33.5–56.75)	26 (15.25–39)	42 (30–67)
**26–35**	52 (32–92)	88 (47.5–>200)	52 (36–72)	56 (33–65.5)	57 (38–94.25)
**36–45**	48 (30.5–101.5)	36.5 (23.5–59)	39 (23.75–76.25)	52.5 (28.25–86.75)	41 (27–83)
**46–55**	79 (31–129.75)	37.5 (23.25–67.25)	40 (24–62)	57 (26–119)	42.5 (26–97)
**56–65**	47 (40–87)	35 (27.5–51.5)	35 (28–56)	48 (36.5–116)	45 (28.25–69.25)
**Total for season**	52 (31.5–99.5)	42 (27.25–80.25)	42 (28–68)	50.5 (27.75–92)	47 (28–88)

**Table 3:** Distribution of BDs’ pre-disinfection microbial load (median, Q1 and Q3 CFU/Plate) by age group and season.

Age Groups	Winter	Spring	Summer	Autumn	Total for Age Groups
	CFU Median (Q1–Q3)	CFU Median (Q1–Q3)	CFU Median (Q1–Q3)	CFU Median (Q1–Q3)	CFU Median (Q1–Q3)
Male donors					
18–25	43 (31.25–52.75)	60 (40.5–108.75)	47 (38–60)	21.5 (17.25–25.75)	42 (34–63.5)
26–35	52 (38–88)	108 (63–>200)	53.5 (32–79.5)	41 (28–58)	59 (38.75–98.5)
36–45	48 (35–101.5)	36.5 (23.5–59)	57 (24.5–84.5)	54.5 (29.75–86.75)	44.5 (27–84)
46–55	79.5 (31–100)	42 (24–67)	40 (28–62)	47.5 (26–98.75)	42 (26–93.5)
56–65	47 (39.25–72)	29.5 (23–42)	32 (28–56)	49 (38–116)	41 (28–64)
Total for season	50 (34.5–95)	42 (26–81)	44.5 (28–68)	45.5 (27.25–91)	46 (28–88)
Female donors					
18–25	-	>200 (ND)	7 (ND)	32 (19.75–59)	32 (15.25–93)
26–35	58.5 (28.75–89.75)	47 (41.5–124)	52 (48–72)	57 (42.5–81.25)	54 (37.5–90.75)
36–45	46 (28.75–117)	39 (27.25–50.25)	37 (24–41)	47 (23.5–81)	41 (28–67)
46–55	56.5 (28.5–>200)	33 (21–65)	41.5 (14–86.25)	62 (38–>200)	47.5 (24–>200)
56–65	>200 (ND)	47.5 (35–162.75)	44 (28.25–56)	47 (32–100.5)	47.5 (32–108.25)
Total for season	61 (28.5–192)	47 (31–68)	41 (22–59)	54.5 (28.75–104.75)	47 (28–96)

ND = not determined. Single value (single donor) by age group in the season.

**Table 4:** Results of kernel nonparametric regression between pre-disinfection skin microbial load and age, season, and sex.

Pre-Disinfection	Observed Estimate	Bootstrap Std. Err.	z	*p*-Value	95% Confidence Interval	
**Mean Microbial load**	71.47405	3.985308	17.93	0.000	64.38593	79.83375
**Effect**						
age	0.0350103	0.3307199	0.11	0.916	−0.6965035	0.6905527
**Season**						
(2 vs. 1)	3.454162	7.549786	0.46	0.647	−12.66011	18.50325
(3 vs. 1)	10.29354	9.047116	1.14	0.255	−9.004311	27.59761
(4 vs. 1)	14.78694	11.39795	1.30	0.195	−7.863455	36.94938
**Sex**						
(F vs. M)	7.008879	8.498945	0.82	0.410	−9.287401	23.64976

1 = summer; 2 = spring; 3 = autumn; 4 = winter.

**Table 5:** Loss of disinfection effectiveness as a function of microbial load: trend of proportions.

Pre-Disinfection Quartiles * (a)	No. of Positive Plates ** (b)	Proportion (b/a)	Chi-Squared for Trend	*p*-Value
1st (n. = 82)	4	0.049	16.801	0.0000
2nd (n. = 83)	10	0.120		
3rd (n. = 82)	13	0.159		
4th (n. = 83)	23	0.277		

* Pre-disinfection skin microbial load ordered into quartiles (83 observations per quartile). ** at least 1 CFU/plate post-disinfection.

## Data Availability

The data presented in this study are available on reasonable request from the corresponding author.
